# Evaluation and Genome Analysis of *Bacillus subtilis* YB-04 as a Potential Biocontrol Agent Against *Fusarium* Wilt and Growth Promotion Agent of Cucumber

**DOI:** 10.3389/fmicb.2022.885430

**Published:** 2022-06-09

**Authors:** Wen Xu, Qian Yang, Fan Yang, Xia Xie, Paul H. Goodwin, Xiaoxu Deng, Baoming Tian, Lirong Yang

**Affiliations:** ^1^Henan International Joint Laboratory of Crop Protection, Henan Biopesticide Engineering Research Center, Institute of Plant Protection Research, Graduate T&R Base of Zhengzhou University, Henan Academy of Agricultural Sciences, Zhengzhou, China; ^2^School of Agricultural Sciences, Zhengzhou University, Zhengzhou, China; ^3^Institute of Horticulture, Henan Academy of Agricultural Sciences, Zhengzhou, China; ^4^School of Environmental Sciences, University of Guelph, Guelph, ON, Canada

**Keywords:** *Fusarium oxysporum* f.sp. *cucumerinum*, biocontrol agent, genome sequencing and assembly, *Bacillus subitilis*, growth promotion

## Abstract

Cucumber wilt caused by *Fusarium oxysporum* f.sp. *cucumerinum* (*Foc*) is a highly destructive disease that leads to reduced yield in cucumbers. In this study, strain YB-04 was isolated from wheat straw and identified as *Bacillus subtilis.* It displayed strong antagonistic activity against *F. oxysporum* f.sp. *cucumerinum* in dual culture and exhibited significant biocontrol of cucumber *Fusarium* wilt with a higher control effect than those of previously reported *Bacillus* strains and displayed pronounced growth promotion of cucumber seedlings. *B. subtilis* YB-04 could secrete extracellular protease, amylase, cellulose, and β-1,3-glucanase and be able to produce siderophores and indole acetic acid. Inoculation with *B. subtilis* YB-04 or *Foc* increased cucumber defense-related enzyme activities for PPO, SOD, CAT, PAL, and LOX. However, the greatest increase was with the combination of *B. subtilis* YB-04 and *Foc*. Sequencing the genome of *B. subtilis* YB-04 showed that it had genes for the biosynthesis of various secondary metabolites, carbohydrate-active enzymes, and assimilation of nitrogen, phosphorous, and potassium. *B. subtilis* YB-04 appears to be a promising biological control agent against the *Fusarium* wilt of cucumber and promotes cucumber growth by genomic, physiological, and phenotypic analysis.

## Introduction

Cucumber (*Cucumis sativus* L.) is an important vegetable crop worldwide. Cucumber wilt caused by *Fusarium oxysporum* f.sp. *cucumerinum* (*Foc*) is one of the most destructive diseases of cucumber that can lead to severe losses in yield and quality (Zhou et al., 2017). *Foc* enters root tissues by direct penetration or wounds causing visible symptoms, including necrotic lesions, vascular and root wilt, and ultimately death (Ahn et al., 1998). It can survive up to 20 years in soil (Zhao et al., 2017). Furthermore, there are no commercially available cucumber cultivars with resistance against *Fusarium* wilt. Therefore, biological control of cucumber *Fusariu*m wilt using antagonistic microorganisms has been considered to be a promising alternative.

Beneficial microorganisms can be used as biological control agents (BCAs) against different plant diseases. Many of these are species of *Bacillus*, which are also plant growth-promoting bacteria (PGPB) that can improve plant growth by producing secondary metabolites, such as fengycin, surfactin, bacillaene, and macrolactin, siderophores, and indole acetic acid (IAA) and secreting hydrolases to suppress plant pathogens and promote plant growth (Blake et al., 2021). More importantly, *Bacillus* species have several advantages, such as rapid growth, spore production, safe, non-pathogenic nature, and adaptation to broader environmental conditions (Nayak, 2021). However, there are several problems in the field application of microbial agents, including lack of high-efficiency biocontrol, relatively short shelf life, and variable control effectiveness. Therefore, understanding the growth-promotion and biocontrol mechanisms of beneficial microorganisms can significantly contribute to improved application efficacy of BCAs.

One way to better understand these organisms is through whole genome sequencing allowing for the discovery of genes for the production of bioactive compounds responsible for biocontrol and growth promotion (Bauman et al., 2021). For instance, sequencing the complete genome of the BCA *Bacillus velezensis* 9912D revealed gene clusters for secondary metabolite synthesis, including several potentially new lantibiotics (Pan et al., 2017). Similarly, the complete genome of *Bacillus subtilis* 7PJ-16 revealed genes for biosynthesis of antimicrobial metabolites and promoting plant growth traits, indicating its ability to act as a BCA and PGPB (Xu et al., 2019). The genome of *B. subtilis* 9407 showed that it had genes for the biocontrol mechanism against bacterial fruit blotch, including genes for a newly identified subtilosin A, bacilysin, and bacillaene (Gu et al., 2021).

In this study, strain YB-04 was isolated from wheat straw. A number of BCA and PGPB traits were screened for YB-04 in culture. The genome of strain YB-04 was sequenced to identify a number of genes associated with BCA and PGPB traits. Plant growth promotion of cucumber seedlings by soil inoculation of YB-04 was assessed based on chlorophyll content and growth of shoots, roots, stems, and leaves. Biocontrol activity by YB-04 against *Fusarium* wilt of cucumber was assessed based on disease severity and disease index. Furthermore, the activities of cucumber defense-related enzymes activities, both by YB-04 alone and in combination with *Foc* inoculation, were examined. The discovery and characterization of *B. subtilis* YB-04 indicate that it is a promising BCA and PGPB of cucumber.

## Materials and Methods

### Isolation of Strain YB-04 and *in vitro* Antagonism Test

Strain YB-04 was isolated from wheat straw and cultured in LB broth by a dilution plate method at 37°C, collected from a field (E113°97’, N35°05’) at the Henan Academy of Agricultural Sciences in Xinxiang, Henan, China in June. *Foc* was obtained from the College of Plant Protection, Henan Agricultural University. Antagonistic activity against *Foc* was performed by a dual culture where *Foc* was grown on PDA at 28°C for 5 days, and then 5 mm agar plugs were excised and transferred to the center of another PDA plate. Strain YB-04 was placed 3 cm away from the edge of the Petri dish, and the growth rate of *Foc* was measured relative to the control, which was *Foc* without strain on a plate (Xu et al., 2020).

### Biocontrol Efficiency of Strain YB-04 Against Cucumber *Fusarium* Wilt and Growth Promotion on Cucumber Seedlings

Strain YB-04 was cultured in LB broth for 24 h at 37°C with shaking at 180 rpm and harvested by centrifugation (4,000 × *g* for 5 min), washed once with LB broth, and adjusted to 10^8^ CFU/ml based on OD at 595 nm. *Foc* was grown on PDA at 28°C for 5 days, and then ten 5 mm agar plugs were excised and transferred to 100 ml PDB. The broths were incubated at 28°C in a shaker at 180 rpm for 3 days. The *Foc* cultures were filtered through 4 layers of sterile gauze, and the filtered spores were adjusted to 10^5^ spores/ml using a hemacytometer (XB-K-25, Qiujing, Shanghai, China).

Cucumber seeds of cultivar Chuancui No. 3 were surface-sterilized in 75% ethanol (v/v) for 30 s and then rinsed with sterile water three times. The seeds were air-dried and each seed was planted in a separate pot (10 cm high, 10 cm diameter) filled with a 400 g sterilized mixture of soil. The plants were grown in the greenhouse at 25°C with a 16 h light/8 h dark photoperiod. After 10 days, each pot of cucumber seedlings was treated as follows: (1) drenching with 15 ml of YB-04 suspension; (2) first drenching with 15 ml l of YB-04 suspension and 24 h of later drenching with 15 ml *Foc* spore suspension; (3) first drenching with 15 ml of 0.1% hymexazol and 24 h later drenching with 15 ml *Foc* spore suspension; (4) drenching with 15 ml of sterile distilled water; or (5) drenching with 15 ml of sterile distilled water and 24 h later drenching with 15 ml of *Foc* spore suspension. Each treatment was performed using 12 plants with 3 replicates. At 20 and 45 days post inoculation (dpi) with YB-04, chlorophyll content, shoot height and fresh weight, root length and fresh weight, stem thickness, and leaf area were measured, and disease severity and disease index were recorded for plants inoculated with *Foc* at 45 days post YB-04 inoculation (Chen et al., 2010). In brief, disease severity was assessed using a 0–4 disease scale; 0 = leaf asymptomatic; 1 = leaf wilting below 1/4 of cucumber seedling; 2 = leaf wilting in 1/4 to 1/2 of cucumber seedling; 3 = leaf wilting above 1/2 of cucumber seedling; 4 = the whole plant was wilted and died. The disease index was calculated using DI = [[(0 × N0) + (1 × N1) + (2 × N2) + (3 × N3) + (4 × N4)]/T × 4] × 100, where N is the number of cucumber seedlings for each disease score and T is the total number of cucumber seedlings. Disease incidence = [N1 + N2 + N3 + N4]/T × 100%. Control efficacy = (DI of control - DI of treatment)/DI of control × 100%. The chlorophyll content of leaves was measured by using a SPAD-502 Plus chlorophyll content meter (Konica Minolta, Tokyo, JP). Root length and shoot height were measured with a ruler. The fresh weight of root and shoot was recorded with an analytical balance (ME203E, Mettler Toledo, Shanghai, China). Stem thickness was measured at 2 cm from the crown with a vernier caliper (MNT-200, Shanghai Meinaite Metals Instruments Co., Shanghai, China).

### Determination of Defense Enzyme Activities in the Cucumber Leaves

After 20 days post YB-04 inoculation, leaves were harvested and stored at –80°C. In brief, 0.5 g leaves were ground in liquid nitrogen, and 1 ml of extraction buffer was added. After centrifugation at 8,000 × *g* for 10 min, the supernatant was removed for enzyme assays. Enzyme activities were measured using assay kits for PPO (Cat. No. BC0195), SOD (Cat. No. BC0175), CAT (Cat. No. BC0205), PAL (Cat. No. BC0215), and LOX (Cat. No. BC0325) following the procedures of the manufacturer (Solarbio, Beijing, China). Absorbance was determined by using a plate reader (Tecan Spark, Tecan, Switzerland).

### Detection of Plant Growth-Promoting Bacteria and Biological Control Agents Traits

Protease activity was detected with single colonies of YB-04 grown at 30°C for 5 days on skim milk agar (0.1 g CaCl_2_, 5.0 g NaCl, 10.0 g skim milk, 10.0 g peptone, and 18.0 g of agar per liter, pH 7.2). Protease activity was observed as clear zones around the colonies (Kazanas, 1968). Amylase activity was detected with single colonies grown at 30^°^C for 48 h on starch agar (10.0 g soluble starch, 10.0 g tryptone, 5.0 g glucose, 5.0 g NaCl, 5.0 g beef extract, and 18.0 g of agar per liter, pH 7.2). Lugol’s iodine solution (1% iodine in 2% potassium iodide w/v) was added to the starch agar plate, and amylase activity was observed as a colorless halo (Al-Naamani et al., 2015). Cellulose activity was assayed with single colonies grown for 7 days at 30°C on carboxymethylcellulose agar (5.0 g CMC-Na, 0.1 g MgSO_4_⋅7H_2_O, 0.25 g (NH_4_)_2_SO_4_, 0.25 g K_2_HPO_4_, and 18.0 g of agar per liter, pH 5.5). The plates were flooded with 1% (m/v) Congo Red, and then washed with sterilized distilled water, and cellulose activity was detected as a clear zone (Teather and Wood, 1982). The β-Glucanase activity was assayed with single colonies grown at 30^°^C for 2 days on β-glucan agar (0.05 g glucose, 0.5 g yeast extract, 1 g peptone, 0.5 g NaCl, 0.01 g Congo Red, and 18.0 g of agar per liter, pH 7.0). The β-Glucanase activity was indicated by a clear zone around the colonies (Teather and Wood, 1982). Siderophore production was determined with single colonies grown at 30^°^C for 2 days in the dark on Chrome Azurol S blue agar (10 ml 20% sucrose solution, 30 ml 10% acid hydrolyzed casein, 1 ml 1 mmol/L CaCl_2_, 5 ml 0.1 mol/L phosphate-buffered saline (pH 6.8), 50 ml CAS dyeing solution, and 18 g of agar per liter, pH 7.2). Siderophore production was indicated by a change from blue to orange around the colonies (Schwyn and Neilands, 1987). IAA production was measured with single colonies grown at 30^°^C for 2 days on L-tryptophan nutrient broth (3 g beef extract, 10 g peptone, 5 g NaCl,0.5 g L-tryptophan per liter, pH 7.2). After centrifugation at 14,000 × *g* for 10 min,1 ml of supernatant was mixed with 2 ml of Salkowski stain, and then kept at room temperature in the dark for 30 min (Glickmann and Dessaux, 1995). All of the above reagents were of analytical grade and produced by China National Pharmaceutical Group Corp., Shanghai, China.

### Genome Sequencing and Assembly of Strain YB-04

Strain YB-04 was grown in LB broth for 16 h at 37°C by shaking at 180 rpm. Genomic DNA was extracted with a Mini-BEST Bacterial Genomic DNA Extraction Kit Ver. 3.0 following the manufacturer’s instructions (Takara, Beijing, China). An approximately 10 kb insert sequencing library was constructed and sequencing was performed using the PacBio Sequel II system (Pacific Biosciences, Menlo Park, CA, United States) by Frasergen (Wuhan, Hubei, China). Sequencing reads were *de novo* assembled by using HGAP4 (Chin et al., 2013) and the Canu (v.1.6) (Koren et al., 2017) software. The depth of genome coverage was analyzed by using the align tool (BLASR, v0.4.1) (Chaisson and Tesler, 2012). The assembled complete genome sequence was deposited in NCBI GenBank (Accession number CP072525). A circular map of the genome was constructed by using Circos (v0.64) (Krzywinski et al., 2009).

### Genome Annotation of Strain YB-04

The genome of strain YB-04 was annotated using Glimmer (v3.02) (Delcher et al., 2007). The tRNA and rRNA genes were identified by tRNAscan-SE (v2.0) (Lowe and Eddy, 1997) and RNAmmer (v1.2) (Lagesen et al., 2007), respectively. Functional descriptions of putative protein encoding genes were done by BLASTx with an *E*-value threshold of 1e-5 using the NCBI Non-Redundant protein database (NR), Swiss-Prot, Clusters of Orthologous Groups (COG), Kyoto Encyclopedia of Genes and Genomes (KEGG), and Gene Ontology (GO).

### Phylogenetic Relationship of Strain YB-04

The 16S rRNA gene sequences of strain YB-04 and *B. velezensis FZB42*, *B. velezensis* YB-130, *B. subtilis* H1, *B. subtilis* 168, *B. licheniformis* SRCM103583, *B. licheniformis* ATCC 14580, *B. altitudinis* CHB19, *B. altitudinis* GQYP101, *B. pumilus* SF-4, and *B. pumilus* ZB201701 were obtained from the genomes (GenBank IDs: CP000560.2, CP054562.1, CP026662.1, NC_000964.3, CP035404.1, CP034569.1, CP043559.1, CP040514.1, CP047089.1, and CP029464.1, respectively). A tree of the 16S rRNA gene sequences was constructed with MEGA 7.0 using the Neighbor Joining method (Kumar et al., 2016). Average Nucleotide Identity (ANI) was calculated using an ANI calculator (Yoon et al., 2017).

### Analysis of Genes Encoding CAZymes and Gene Clusters Responsible for the Biosynthesis of Secondary Metabolites

Protein-coding genes in the genome of strain YB-04 were aligned with the carbohydrate active enZYme (CAZy) database (Lombard et al., 2014) using dbCAN2 (Zhang et al., 2018) and HMMER (v3.1b2) (Finn et al., 2011) with an *E*-value threshold of 1e-15. Identification of gene clusters for the synthesis of secondary metabolites was analyzed by using antiSMASH5.0 (Blin et al., 2019).

### Statistical Analysis

Statistical analysis was performed using SPSS v21.0 by one-way analysis of variance (ANOVA). Means were compared with Duncan’s multiple range tests at a probability of *p* ≤ 0.05.

## Results

### Isolation of YB-04 and Wilt Disease Biocontrol Activity *in vitro* and *in vivo*

**FIGURE 1:**
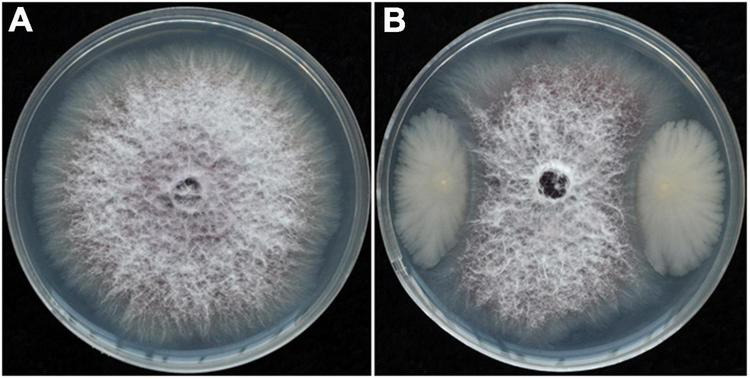
Colony morphology of *Foc* co-cultivated with or without strain YB-04. **(A)** Colony morphology of *Foc* in PDA; **(B)** inhibition of strain YB-04 on *Foc* growth.

**FIGURE 2:**
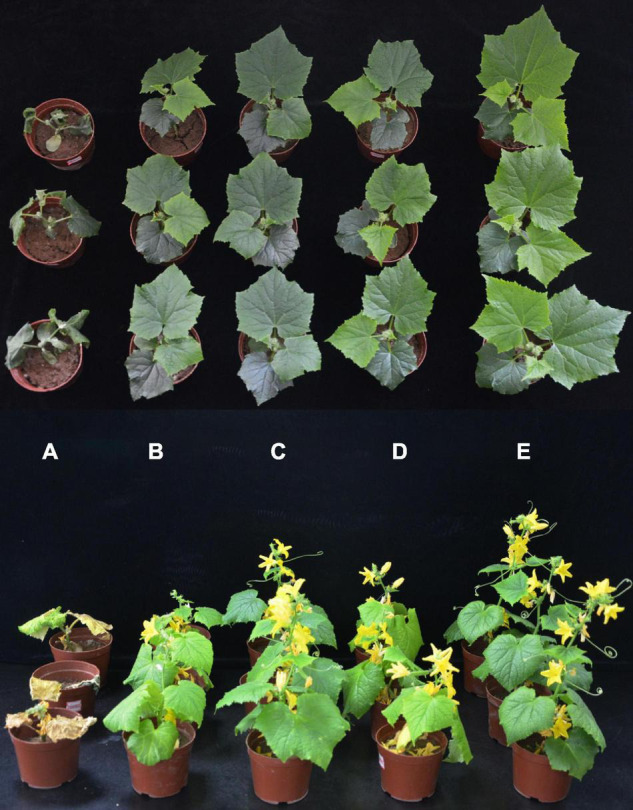
Effect of strain YB-04 against *Fusarium* wilt and on growth-promotion of Cucumber Seedlings. **(A)** Only *Foc* inoculation; **(B)** *Foc* inoculation and hymexazol treatment; **(C)** inoculation of strain YB-04 and *Foc*; **(D)** sterile distilled water; **(E)** only strain YB-04 inoculation.

Dilution plating from surface-sterilized wheat straw yielded numerous colonies with different colony appearances. Twenty strains were purified and screened for antagonistic activity against *Foc* in dual culture and reduced wilt severity of cucumber inoculated with *Foc* in the greenhouse (data not shown). Strain YB-04 was selected based on having the greatest antagonistic activity against *Foc* in culture (Figure 1) and reduced wilt severity of cucumber seedlings at 20 days after *Foc* inoculation (Figure 2). Disease incidence, disease index, and control efficacy at 45 dpi revealed that YB-04 significantly reduced wilt symptoms caused by *Foc* to levels slightly less than the chemical fungicide hymexazol (Table 1).

**TABLE 1:** Disease incidence, disease index, and control efficacy of *B. subtilis YB-04* against cucumber *Fusarium* wilt.

Treatment	Disease incidence (%)	Disease index	Control efficacy (%)
FOC	95.07 ± 0.41a	107.51 ± 0.4a	
FOC + 0.1% Hymexazol	3.48 ± 0.15b	13.24 ± 0.14b	87.68 ± 0.08a
FOC + YB-04	2.42 ± 0.21c	8.69 ± 0.09c	91.92 ± 0.12a

*Data are the mean ± standard deviation (SD); different letters (a–c) in the same column indicate significant difference at p-values < 0.05 level.*

### Growth-Promotion Activity of Strain YB-04

At 20 and 45 dpi with strain YB-04, there was a significant increase in chlorophyll content, height and fresh weight of shoot, root length and fresh weight, stem thickness, and leaf area compared to non-treated cucumber seedlings (Figure 2 and Table 2). At 20 dpi, the greatest increases were observed for the fresh weight of shoots and roots at 115.91 and 334.88%, respectively. At 45 dpi, the greatest increases were observed for shoot height and leaf area at 79.03 and 49.07%, respectively.

**TABLE 2:** Effects of *B. subtilis* YB-04 on growth parameters of cucumber seedlings.

		FOC	FOC + 0.1% Hymexazol	FOC + YB-04	CK	YB-04
20 days after inoculation	Chlorophyll content (SPAD)	36.20 ± 0.52d	42.13 ± 0.23c	50.97 ± 0.30a	41.87 ± 0.39c	44.17 ± 0.41b
	Shoot height (cm)	6.47 ± 0.20c	12.27 ± 0.27b	16.27 ± 0.23a	11.80 ± 0.17b	16.63 ± 0.09a
	Stem thickness (mm)	3.47 ± 0.03e	4.33 ± 0.03c	4.47 ± 0.01b	3.74 ± 0.03d	4.60 ± 0.05a
	Shoot fresh weight (g)	2.11 ± 0.02e	7.17 ± 0.04c	9.66 ± 0.08b	4.84 ± 0.06d	10.45 ± 0.07a
	Root length (cm)	7.81 ± 0.06e	19.50 ± 0.42c	21.30 ± 0.32 b	18.50 ± 0.35d	35.27 ± 0.27a
	Root fresh weight (g)	0.16 ± 0.02e	1.34 ± 0.05c	1.72 ± 0.05 b	0.43 ± 0.02d	1.87 ± 0.03a
	Leaf area (cm^2^)	53.54 ± 0.70 e	74.37 ± 0.51c	84.53 ± 1.28b	71.30 ± 0.66d	103.12 ± 0.91a
45 days after inoculation	Chlorophyll content (SPAD)	10.73 ± 10.73d	30.90 ± 30.90c	41.27 ± 0.64a	34.30 ± 0.61b	42.17 ± 0.64a
	Shoot height (cm)	9.03 ± 0.24d	16.21 ± 0.52c	26.27 ± 0.69b	17.50 ± 0.69c	31.33 ± 0.66a
	Stem thickness (mm)	4.11 ± 0.06c	4.43 ± 0.02b	4.52 ± 0.02b	4.20 ± 0.04c	4.94 ± 0.03a
	Shoot fresh weight (g)	5.06 ± 0.02e	8.57 ± 0.76d	14.14 ± 0.58b	12.42 ± 0.30c	15.91 ± 0.24a
	Root length (cm)	13.02 ± 0.26d	14.77 ± 0.38c	18.97 ± 0.35b	15.02 ± 0.34c	20.40 ± 0.57a
	Root fresh weight (g)	1.88 ± 0.04d	4.74 ± 0.06c	6.21 ± 0.06a	5.58 ± 0.07b	6.35 ± 0.07a
	Leaf Area (cm^2^)	44.41 ± 1.16e	78.01 ± 0.90d	106.28 ± 1.12b	91.38 ± 1.13c	136.22 ± 0.78a

*Data are the mean ± standard deviation (SD); different letters (a–e) in the same line indicate significant difference at p-values < 0.05 level.*

At 20 and 45 dpi with strain YB-04 and *Foc* inoculation, there was also a significant increase in chlorophyll content, height, and fresh weight of shoot, root length and fresh weight, stem thickness, and leaf area compared to that of the *Foc* inoculated cucumber seedlings (Figure 2 and Table 2). This was also observed with the *Foc* inoculated seedlings treated with hymexazol. However, strain YB-04 treatment of the *Foc*-inoculated seedlings resulted in significantly higher chlorophyll content, shoot height and fresh weight, root length and fresh weight, and leaf area than *Foc*-inoculated seedlings with hymexazol. However, there was no significant difference in the stem thickness of *Foc*-inoculated seedlings with strain YB-04 or hymexazol at 45 dpi.

### Effect of Strain YB-04 on Activities of Defense-Related Enzymes in Cucumber Seedlings

At 20 dpi with strain YB-04, cucumber seedlings showed significantly higher activities of SOD, CAT, PAL, and LOX, but not PPO, compared to non-treated seedlings (Table 3). Inoculation with *Foc* also significantly increased those enzyme activities, except PPO, compared to seedlings without *Foc* inoculation. The highest activities were observed with *Foc* inoculation and strain YB-04 treatment, which was significantly higher for all the enzymes compared to *Foc* inoculation. However, the activities of PAL and CAT significantly increased but LOX, PPO, and SOD significantly decreased with *Foc* inoculation and hymexazol treatment compared to *Foc* inoculation.

**TABLE 3:** Five defense enzyme activities of cucumber leaves under different treatments.

	LOX (U/g)	PAL (U/g)	CAT (U/g)	PPO (U/g)	SOD (U/g)
FOC	1450.70 ± 28.01b	18.76 ± 0.65c	149.34 ± 7.58c	76.82 ± 1.14b	160.15 ± 0.83b
FOC + 0.1% Hymexazol	618.12 ± 9.48c	26.54 ± 0.57b	180.74 ± 4.61b	43.30 ± 0.75c	141.44 ± 1.19d
FOC + YB-04	3620.26 ± 27.82a	29.90 ± 0.92a	221.38 ± 3.38a	187.93 ± 3.85a	188.54 ± 1.05a
CK	460.10 ± 11.12d	12.64 ± 0.70e	104.99 ± 2.89e	21.72 ± 1.06d	102.22 ± 0.99e
YB-04	650.55 ± 12.38c	15.45 ± 0.63d	119.77 ± 2.48d	77.61 ± 0.62b	155.95 ± 0.97c

*Data are the mean ± standard deviation (SD); different letters (a–e) in the same column indicate significant difference at p-values < 0.05 level.*

### Detection of *in vitro* Antifungal and Growth-Promoting Traits

**FIGURE 3:**
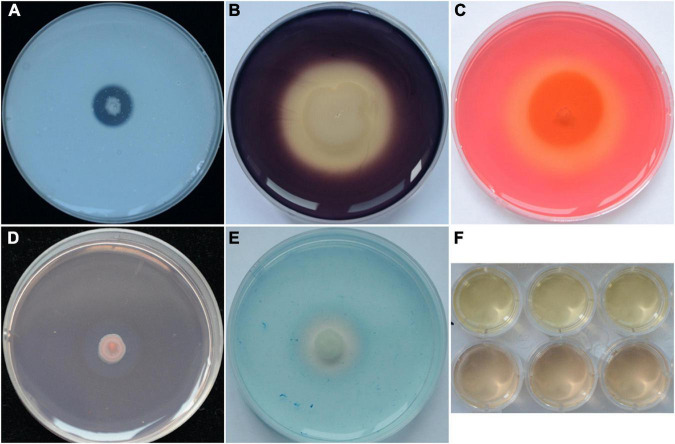
Antifungal and PGP traits of strain YB-04. **(A)** Protease production; **(B)** amylase production; **(C)** cellulose production; **(D)** β-1,3-glucanase production; **(E)** siderophore production; **(F)** IAA production.

Strain YB-04 could secrete protease (Figure 3A), amylase (Figure 3B), cellulose (Figure 3C), and β-1, 3-glucanase (Figure 3D). In addition, it could produce siderophores (Figure 3E) and indole acetic acid (Figure 3F).

### Genome Sequencing, Assembly, and Identification of Strain YB-04

**FIGURE 4:**
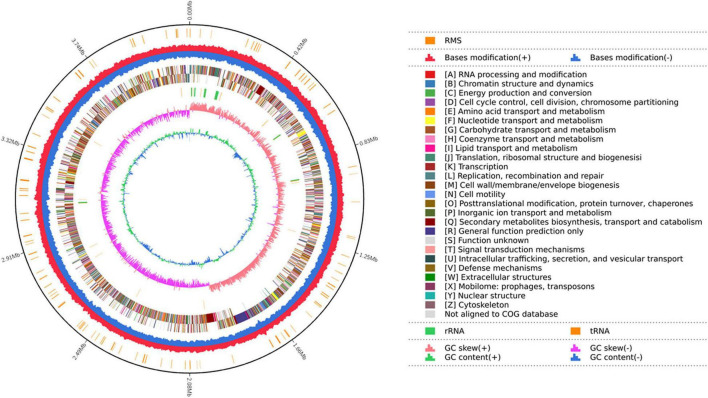
Map of the strain YB-04 genome. The distributions of circles from outwards to inwards are as follows: ring 1 for genome size (black line); ring 2 for the restriction modification system, for forward strand (red) and reverse strand (blue); ring 3 for COG classifications of protein-coding genes on the forward strand and reverse strand; ring 4 for the distribution of tRNAs (brown) and rRNAs (green); ring 5 for GC skew; ring 6 for GC content.

A total of 387,797 high-quality sequencing long reads with a mean length of 10,962 bp and an N50 of 13,374 bp were generated from the genomic DNA of strain YB-04 by the Pacbio sequencing platform. Total base pairs were 4,251,215,058 bp with an 882.47X genome coverage. The YB-04 genome consisted of a single circular chromosome of 4,156,177 bp with a GC content of 43.83% (Figure 4). There were 4,325 protein-coding genes covering 88.62% of the genome with an average gene length of 851.6 bp, which included 87 tRNAs, 30 rRNAs (5S, 16S, 23S), and 22 sRNAs. Four gene islands, three CRISPRs, and four prophages were detected (Supplementary Tables 1–3). For the predicted protein encoding genes, 99.70, 90.73, 75.45, 64.30, and 53.87% could be annotated with the NR, Swiss-Prot, COG, GO, and KEGG databases, respectively (Supplementary Table 4).

**FIGURE 5:**
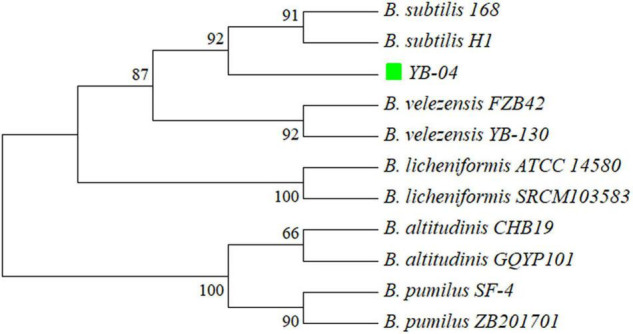
Phylogenetic tree of *B. subtilis* YB-04 and 10 other *Bacillus* species based on 16S rRNA sequences.

**FIGURE 6:**
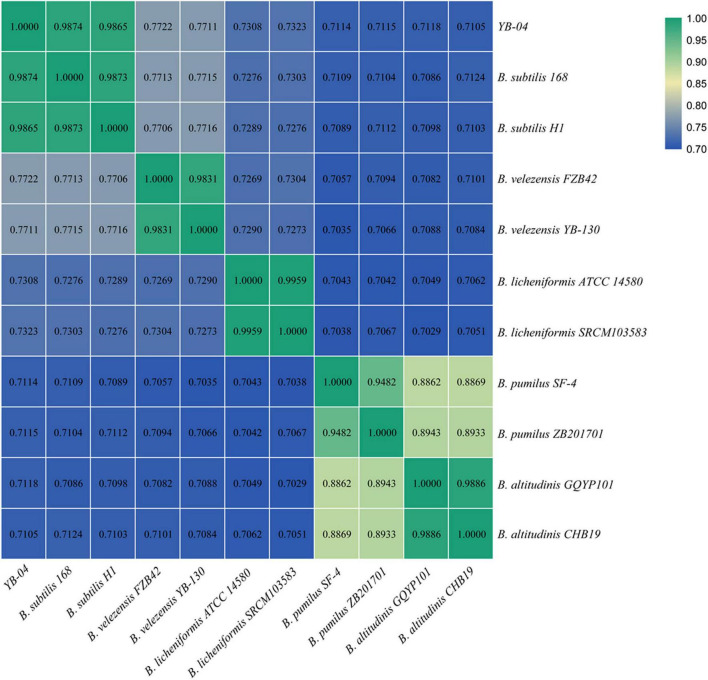
ANI analysis of *B. subtilis* YB-04 and 10 other *Bacillus* species.

A phylogenetic tree based on 16S rRNA gene sequences of strain YB-04 and 10 other *Bacillus* isolates showed that strain YB-04, *B. subtilis* 168, and *B. subtilis* H1 clustered (Figure 5). Strain YB-04 and *B. subtilis* 168 had the maximum ANI value of 98.74%, followed by B. *subtilis* H1with 98.65%, which is higher than the cutoff of 95–96% for bacterial species identity. ANI values between strain YB-04 and the 8 other *Bacillus* species ranged from 71.05 to 77.22% (Figure 6). Therefore, strain YB-04 was identified as *B. subtilis*.

### Genome Analysis of Selected Genes of *Bacillus subtilis* YB-04

**FIGURE 7:**
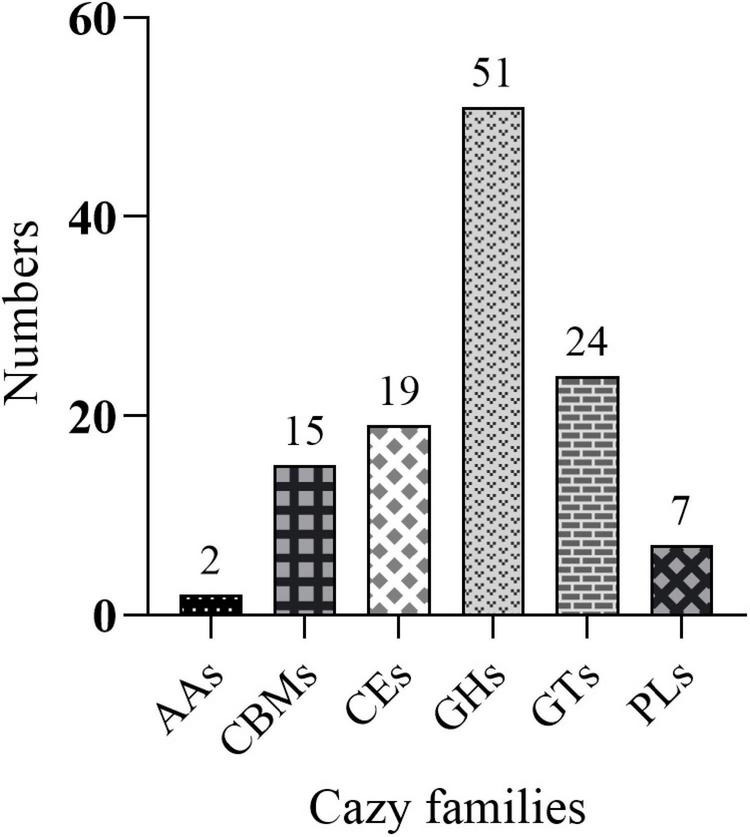
Distribution of CAZy families in the genome of *B. subtilis* YB-04.

The genome of *B. subtilis* YB-04 had 111 genes identified as putative CAZymes, namely, 2 auxiliary activities (AAs), 7 polysaccharide lyases (PLs), 15 carbohydrate-binding modules (CBMs), 19 carbohydrate esterases (CEs) 24 glycosyltransferases (GTs), and 51 glycoside hydrolases (GHs) (Figure 7 and Supplementary Table 5). Six of those were classified as both GHs and CBMs.

There were 13 gene clusters predicted to be responsible for the biosynthesis of secondary metabolites. At 100% similarity, there was each matching gene clusters for bacillaene, fengycin, bacillibactin, subtilin, subtilosin A, and bacilysin synthesis. There was also one gene cluster with 82% similarity to that for surfactin synthesis. There were 6 biosynthetic gene clusters with no similarity in the antiSMASH database that appeared to be novel biosynthetic gene clusters of secondary metabolites. Based on their matches to the antiSMASH database, these were one gene cluster each for types of lanthipeptide-class-i, Type III PKS, tRNA-dependent cyclodipeptide synthases, other unspecified ribosomally synthesized, post-translationally modified peptide products, and two gene clusters, each encoding for terpenes (Table 4).

**TABLE 4:** List of the putative gene clusters encoding for secondary metabolites by antiSMASH in the *B. subtilis* YB-04 genome.

Clusters	Types	Genomic locations	Most similar known clusters	Similarity
Cluster 1	NRPS	349,833–413,272	Surfactin	82%
Cluster 2	Terpene	1,124,835–1,145,348		
Cluster 3	Lanthipeptide-class-i	1,699,549–1,725,648		
Cluster 4	transAT-PKS,PKS-like,T3PKS,transAT-PKS-like,NRPS	1,747,915–1,862,664	Bacillaene	100%
Cluster 5	NRPS, betalactone	1,920,534–2,002,654	Fengycin	100%
Cluster 6	Terpene	2,073,306–2,095,204		
Cluster 7	T3PKS	2,142,940–2,184,037		
Cluster 8	NRPS	3,179,833–3,229,574	Bacillibactin	100%
Cluster 9	lanthipeptide-class-i	3,377,840–3,404,065	Subtilin	100%
Cluster 10	CDPS	3,523,308–3,544,054		
Cluster 11	Sactipeptide	3,768,784–3,790,395	Subtilosin A	100%
Cluster 12	Other	3,797,486–3,838,904	Bacilysin	100%
Cluster 13	RiPP-like	4,040,385–4,053,116		

The genome of *B. subtilis* YB-04 contained predicted genes for an ATP-dependent phosphate uptake system *PstABCS*, phoPR operon for regulating Pho regulon in response to phosphate limitation, and alkaline phosphatase genes of *phoA* and *phoD* for phosphorus acquisition (Table 5). It also contained the *nasABCDEF* gene cluster for nitrite transport and reduction. Additionally, there were the potassium uptake system *ktrABCD*, a putative gamma-glutamylcyclotransferase *YkqA* for potassium assimilation, and a putative potassium efflux channel *yugO*.

**TABLE 5:** Genes responsible for nitrogen, phosphorous, and potassium assimilation identified in the strain YB-04 genome.

Function	Gene	UniProt accession No.	Description	Best hit in YB-04	Identity
Phosphate assimilation	*phoA*	P19406	Alkaline phosphatase 4	orf00986	99.35%
	*phoR*	P23545	Alkaline phosphatase synthesis sensor protein PhoR	orf03027	99.48%
	*phoP*	P13792	Alkaline phosphatase synthesis transcriptional regulatory protein PhoP	orf03028	99.58%
	*phoD*	P42251	Alkaline phosphatase D	orf00275	99.49%
Phosphate transport	*pstS*	P46338	Phosphate-binding protein	orf02500	99.67%
	*pstC*	A0A6M4JLF7	Phosphate transport system permease protein	orf02499	99.35%
	*pstB1*	P46342	Phosphate import ATP-binding protein PstB 1	orf02496	99.62%
	*pstB2*	P46341	Phosphate import ATP-binding protein PstB 2	orf02497	99.63%
	*pstA*	A0A6M3ZE53	Phosphate transport system permease protein	orf02498	100.00%
Nitrate/nitrite assimilation	*nasD*	P42435	Nitrite reductase	orf00344	99.26%
	*nasE*	P42436	Assimilatory nitrite reductase [NAD(P)H] small subunit	orf00343	100.00%
	*nasA*	P42432	Nitrate transporter	orf00347	99.50%
	*nasC*	P42434	Assimilatory nitrate reductase catalytic subunit	orf00345	98.03%
	*nasB*	P42433	Assimilatory nitrate reductase electron transfer subunit	orf00346	97.54%
	*nasF*	P42437	Uroporphyrinogen-III C-methyltransferase	orf00342	97.30%
Potassium assimilation	*ktrC*	P39760	Ktr system potassium uptake protein C	orf01561	100.00%
	*ykqA*	P39759	Putative gamma-glutamylcyclotransferase YkqA	orf01560	96.75%
	*ktrD*	O31658	Ktr system potassium uptake protein D	orf01445	100.00%
	*yugO*	Q795M8	Putative potassium channel protein YugO	orf03270	100.00%
	*ktrB*	O32081	Ktr system potassium uptake protein B	orf03241	99.10%
	*ktrA*	O32080	Ktr system potassium uptake protein A	orf03240	99.10%

## Discussion

*Fusarium* wilt disease caused by *Foc* is one of the most devastating soil-borne diseases of cucumber, resulting in severe yield losses throughout the world (Zhou et al., 2018). While the use of BCAs to control plant diseases and PGPBs to improve plant growth is considered to be promising (Radhakrishnan et al., 2017; Koskey et al., 2021), new strains are needed to screen for greater efficiency, shelf life, and consistency. As part of that, an in-depth analysis of growth promotion and biocontrol traits will help in developing them into successful microbial products.

In this study, *B. subtilis* YB-04 was found to be a BCA with antagonistic activity against *Foc* in dual culture and significantly reduced *Fusarium* wilt caused by *Foc* at levels comparable to hymexazol, which is used to control the disease in China. It was also a PGPB with pronounced growth promotion on cucumber seedlings. Previously, *B. subtilis* B579, *B. subtilis* MBI600, and *B. subtilis* B068150 were also shown to significantly reduce cucumber *Fusarium* wilt and promote cucumber growth (Chen et al., 2010; Li et al., 2012; Samaras et al., 2020). Compared to those bacteria, the percentage reduction in the *Fusarium* wilt of cucumber by *B. subtilis* YB-04 was greater than those achieved by *B. subtilis* B068150, *B. subtilis* B579, or *B. subtilis* MBI600. The percentage of increased growth-promotion based on the shoot and root fresh weight and plant height by *B. subtilis* YB-04 was greater than those achieved by *B. subtilis* B579 or *B. subtilis* MBI600. Thus, *B. subtilis* YB-04 appears to be more effective as a BCA and PGPB than some of the previously described *B. subtilis* tested on cucumber.

To act as a BCA against plant pathogenic fungi, bacteria possess a number of mechanisms including synthesis of hydrolytic enzymes, production of antibiotics, and induction of systemic resistance (Morales-Cedeño et al., 2021; Saeed et al., 2021; Xu et al., 2021). In this study, *B. subtilis* YB-04 had all of those mechanisms. Hydrolytic enzyme activities including protease, amylase, cellulose, and β-1, 3-glucanase were present in pure cultures, and they can break down chitin, glucans, and other polymers in fungal cell walls, thus inhibiting the growth of fungal pathogens (Naglot et al., 2015; Li et al., 2019). Other *B. subtilis* BCAs with similar enzymes include *B. subtilis* BCC6327, *B. subtilis* ZIM3, and *B. subtilis* LR1 (Thakaew and Niamsup, 2013; Banerjee et al., 2017; Dai et al., 2020). Furthermore, a large number of CAZyme genes were found in the genome of *B. subtilis* YB-04, also suggesting that it has a strong capability to be antagonistic against fungal plant pathogens based on the potential degradation and use of fungal polymers as nutrient sources (Banani et al., 2015; Chen et al., 2018; Sui et al., 2020). In addition, gene clusters were found to be responsible for the biosynthesis of known secondary metabolites, including bacillaene, fengycin, bacillibactin, subtilin, subtilosin A, bacilysin, and surfactin, indicating antibiotic production by *B. subtilis* YB-04, which is common in *Bacillus* species (Xu et al., 2019; Su et al., 2020). Other *B. subtilis* BCAs found to produce antibiotics or have genes for antibiotic production included *B. subtilis* BSD-2 for lanthipeptide (Liu et al., 2016). Surfactin and fengycin can also be elicitors of induced systemic resistance in plants (Romero et al., 2007; Ongena et al., 2010). Many studies have reported that plant defense enzymes play important roles in disease resistance (Prasannath, 2017; Ji et al., 2020; Xu et al., 2021). Induction of the activities of defense-related enzymes in leaves following soil inoculation with *B. subtilis* YB-04 and *Foc* indicates a form of systemic resistance. Defense-related enzymes activities for PPO, SOD, CAT, PAL, and LOX could be induced by inoculation with *B. subtilis* YB-04 or *Foc* alone and the greatest increase was with the combination of *B. subtilis* YB-04 and *Foc*. Other *B. subtilis* BCAs causing host induction of defense-related enzyme activities include *B. subtilis* B579, *B. subtilis* SL-44, and *B. subtilis* CBR05 (Chen et al., 2010; Chandrasekaran and Chun, 2016; Wu et al., 2019).

All the growth parameters of cucumber seedlings measured in this study were increased with *B. subtilis* YB-04 treatment. Importantly, growth parameters were all increased much more in infected seedlings with *Foc* treated with *B. subtilis* YB-04 than those infected with *Foc* and treated with hymexazol. This indicates that *B. subtilis* YB-04 can improve plant growth while providing disease control, which would be an advantage over using hymexazol that did not promote growth. This was similar to *Pseudomonas aeruginosa* CQ-40 that controlled tomato gray mold caused by *Botrytis cinerea* and promoted the growth of tomato seedlings, whereas pyrimethanil only controlled the disease with a prevention effect of up to 64.71% (Wang et al., 2020).

To act as a PGPB, bacteria have a variety of mechanisms including the production of enzymes and siderophores for nutrient acquisition and phytohormones to promote growth, and enzymes to reduce the negative effects of various abiotic stresses (Glick, 2012; Saeed et al., 2021). *B. subtilis* YB-04 produced siderophores that can improve iron uptake and alleviate harmful effects of iron on plants that have been associated with enhanced plant growth (Haas, 2003; Dimkpa et al., 2009). The genome of *B. subtilis* YB-04 also contained genes responsible for nitrogen, phosphorous, and potassium assimilation. Plant growth and development depend on macronutrients, such as nitrogen, phosphorous, and potassium, that are mostly obtained from the soil and can be made more available to plants by soil microbes that have the ability to solubilize nutrients and transfer them to plants (Glick, 2012; Rana et al., 2020). Finally, *B. subtilis* YB-04 produced IAA, which may be taken up by the cucumber seedlings stimulating the transcriptional expression of IAA responsive genes and enhancing biomass (Spaepen et al., 2014; Jiang et al., 2020).

In summary, *B. subtilis* YB-04 appears to be an effective BCA against cucumber *Fusarium* wilt and an effective PGPB of cucumber seedlings. The BCA mechanisms could include induced systemic host resistance as indicated by greater host defense-related enzyme activities, and direct pathogen inhibition through secretion of extracellular enzymes and antibiotics. The PGPB mechanisms could include nutrient acquisition *via* siderophores and enzymes for fixing nitrogen and solubilizing potassium and phosphorus, and direct plant growth enhancement through increased amounts of indole acetic acid. Compared to other *B. subtilis* strains used as cucumber *Fusarium* wilt BCAs and PGPBs in cucumber, *B. subtilis* YB-04 is a more effective BCA than all those reported thus far and is a more effective PGPB than most reported so far. Thus, it appears to be a very promising novel beneficial *B. subtilis* strain for cucumber production.

## Data Availability Statement

The datasets presented in this study can be found in online repositories. The names of the repository/repositories and accession number(s) can be found in the article/Supplementary Material.

## Author Contributions

LY and WX conceived the research and designed the experiments. QY, XX, FY, XD, and WX performed the experiments and analyzed the data. WX prepared the manuscript draft. PG, BT, and LY critically revised the manuscript. All authors approved the final version of the manuscript.

## Conflict of Interest

The authors declare that the research was conducted in the absence of any commercial or financial relationships that could be construed as a potential conflict of interest.

## Publisher’s Note

All claims expressed in this article are solely those of the authors and do not necessarily represent those of their affiliated organizations, or those of the publisher, the editors and the reviewers. Any product that may be evaluated in this article, or claim that may be made by its manufacturer, is not guaranteed or endorsed by the publisher.
